# “*Crazy person is crazy person. It doesn’t differentiate*”: an exploration into Somali views of mental health and access to healthcare in an established UK Somali community

**DOI:** 10.1186/s12939-020-01295-0

**Published:** 2020-10-27

**Authors:** Catherine Linney, Siyan Ye, Sabi Redwood, Abdi Mohamed, Abdullahi Farah, Lucy Biddle, Esther Crawley

**Affiliations:** 1 Centre for Academic Child Health, Bristol Medical School, University of Bristol, 1-5 Whiteladies Road, Bristol, BS8 1NU UK; 2 ARC West, Bristol, UK; 3 Golden Key, Bristol, UK; 4 Somali Resource Centre, Bristol, UK; 5 Population Health Sciences, Bristol Medical School, University of Bristol, Bristol, UK

## Abstract

**Background:**

Mental health conditions have been shown to disproportionately affect those from Black, Asian and Minority Ethnic (BAME) communities. Somali communities globally have relatively high levels of mental illness, but low levels of mental health service use, with numerous barriers to care identified.

This study was conducted in an established UK Somali community in the South West of England and aimed to explore community beliefs and views about the causes of mental illness, treatment for mental illness, and access to medical services in general. Participants were asked about how mental health and illness are understood and conceptualised, along with the cultural meaning of mental illness and its manifestations in relation to men, women and young people.

**Design:**

Using a community-based participatory research design, in partnership with local Somali community organisations, the research team conducted four focus groups with a total of 23 participants aged over 18. Open-ended questions were used to facilitate discussion. Transcripts were analysed thematically.

**Results:**

The participants discussed the role of migration and associated stress from the civil war and how that could contribute to mental illness.

Participants tended to view the symptoms of mental illness as physical manifestations such as headaches and to describe a strong community stigma where those with mental health conditions were viewed as “crazy” by others. Barriers to accessing healthcare included language barriers, waiting times and a mistrust of doctors. Various ideas for improvements were discussed, including ideas to reduce stigma and ideas for community initiatives.

**Conclusion:**

Cultural considerations and reducing stigma are vital in improving understanding of mental illness and improving access to mental health services, along with building relationships and trust between the Somali community and health care workers.

## Introduction

Mental health disorders are common in the UK, with around one in six adults (17%) in England meeting the diagnostic criteria for a common mental disorder [1]. In a UK National Health Service (NHS) survey from 2017 one in eight young people under the age of 19 had at least one mental disorder (12.8%) [2] compared to 2004 when one in 10 children had a mental health disorder [2]. Mental disorders are increasingly being diagnosed in the UK; there has been an increase in the prevalence of severe symptoms of common mental disorders in women since the year 2000 [1] (with stable prevalence rates in men) and also a potentially increased recognition of the symptoms of mental illness. Early detection of mental ill-health and intervention can lead to improved outcomes for the patient [3]. Migration and seeking asylum can increase the risk rates of mental illness and there are numerous challenges of addressing migrant and refugee mental health needs [4]. Refugees and migrants have higher prevalence rates of mental illness, with 48.1% of Somali refugees meeting the criteria for Post-Traumatic Stress Disorder (PTSD) [5]. Experiences and difficulties pre, post and during the migratory process all combine to influence mental illness rates [6]. Understanding the knowledge and beliefs communities have of mental illness and the barriers they experience in accessing healthcare is important to ensure those suffering from mental illness can access culturally acceptable and safe treatment [7, 8].

The UK has the largest and longest-established Somali community in Europe [9]. Somali migration to the UK started between the late 1800s and 1960s from Somaliland with migration from Somalia during and after World War II [9]. Since the 1990s, Somali refugees fleeing from Civil War have resettled in the UK [9]. Somali communities in London and the USA have a relatively high level of mental ill-health need but a low level of mental health service use [10, 11]. Somali adolescents also have low rates of service use, but access alternative sources of help (school and religious leaders) more frequently [12]. Higher suicide rates have been anecdotally reported in Somali communities in London, with mental illness a known risk factor for suicide [13].

Previous work in the USA and UK has identified various causes of mental illness in Somali communities, such as stress, worry, fear, loss and spirit possession [10, 14]. Work in the UK, New Zealand and Norway describes coping strategies for mental illness including: religious practices [13–17] and reliance on social networks and religious/ethnic communities [15–17]. There can be an unfamiliarity with Western diagnostic labels and mood disorders in Somali communities, with mental health being divided into categories of “sanity” and “insanity” with those demonstrating violent or uncontrollable behaviours categorised into “insane” [7, 12, 13, 17, 18]. Somali individuals may hide their symptoms or deny that there is an issue, due to a lack of support and social stigma from their community [13, 14]. Little is known about why the Somali community is unable to access mental health services.

Individuals from Black, Asian and Minority Ethnic (BAME) communities are in general less likely to access mental health services. NHS (National Health Service) figures show that White British individuals were the group most likely to access mental health treatment (13.3%) and that Black British adults had the lowest treatment rates (6.2%) [1]. BAME ethnic groups are more likely to be detained under the Mental Health Act compared to White British [19–21] and Black British adults are 3 times more likely to come to the attention of psychosis mental health services through compulsory admission into services than White British [21, 22]. The NHS is currently reviewing the Mental Health Act [23] and has previously tried to improve mental health services for BAME communities in England through various policy initiatives and programs, such as through the Inside Outside report in 2003 [24]. Inside Outside recognised that NHS Mental Health Services were not adequately meeting the needs of BAME users and recommended reform by investing in mental health ‘inside’ NHS services and ‘outside’ in community services to reduce and eliminate inequalities experienced by BAME individuals when accessing mental health services [24]. In the UK, access to mental health is through primary care, with most common mental health problems managed in the GP (General Practitioner) setting [25]. If a referral to specialist mental health services is needed, then the GP is able to refer [26]. Therefore, mental health services in the UK encompasses not only specialist mental health services, but also GPs [14].

In terms of perceptions of mental health services, individuals from African-Caribbean communities in the UK describe experiences and expectations of institutional mistreatment from mental health services and communication difficulties due to misinterpretation of different uses of language and gestures [27]. These can lead to a negative perception of mental health services and to social exclusion and a lack of access to services [27]. A study with BAME individuals in the UK found a perception of power imbalances and perceived discrimination towards BAME individuals, can influence access to mental health services [8].

In terms of general barriers to accessing care, asylum seekers and individuals from BAME (Black, Asian and Minority Ethnic) groups can face numerous barriers to accessing primary care, and therefore receiving a referral to mental health services. These barriers could be due to: previous negative experiences accessing help, English language ability, navigating and understanding the available services (such as registering with a GP surgery, and making appointments), articulating their distress to a GP, and making the decision to access help based on conceptualisation of the symptoms [25, 28]. Additionally Somali communities in London have proposed additional barriers to accessing care including: mistrust of the system and fear, cultural barriers, and anxiety over immigration status and access to housing [13, 14, 28]. Although interpreters may be available, Somali individuals may be concerned about the confidentiality of what the interpreter may witness in the GP appointment [14]. Somali individuals can experience problems registering with a GP practice, or may believe that the GP service can only be accessed for physical health problems and that it is not appropriate to use it for mental health symptoms [25]. Those from Somali communities therefore use A&E departments or other services including specific refugee services for unmet needs [11, 28].

This study aimed to investigate Somali views on mental illness, accessing appropriate healthcare and ideas to improve access and reduce barriers. This study explored Somali community beliefs and views on the causes of mental illness, including how it is conceptualised and cultural factors, perceptions of treatment for mental illness and access to medical services. The questions were asked broadly to encompass views on men, women and children in relation to mental health and access to healthcare. Finally the study aimed to capture the participants’ views on ideas to improve access and to reduce barriers. Individual knowledge of mental health and experiences accessing healthcare services is important, as researchers need to understand population needs to provide adequate and culturally sensitive resources.

## Methods

### Study design

This qualitative focus group project was community driven, as members of the Somali community in Bristol approached the University researchers, via AM and AF, to develop this project in the context of concerns about rising suicide rates in Bristol. The long-term aim of this project is to improve knowledge of mental health and access to mental health services by the Bristol Somali community.

This research was co-produced with two Somali community researchers (AM and AF), utilising a community-based participatory research (CBPR) approach, a methodology used to conduct studies in partnership with community organisations [29]. CBPR utilises mutual respect and co-learning between researchers and community members along with community engagement in a process where community partners contribute their unique strengths to enhance understanding [30–32]. The CBPR methodology chosen for this study combined community members’ experiences and knowledge with the University researchers’ expertise in qualitative research methods [33, 34]. This collaborate approach was used in all aspects of the research design to explore Somali views on mental health and access to healthcare.

Qualitative focus groups were used to explore collective opinions on the subject [35] and provide insights into how beliefs and knowledge operate within the community [36]. Focus groups were selected as they are a useful tool for informing the development of interventions, particularly in multicultural populations [35].

### Setting

The present work is focussed on the Somali population in Bristol, a city in the South West of England. Bristol has a population estimate of 463,400 [37]. In Bristol, the Somali population is the second largest migrant community [37]. ‘Somali’ is not a separate ethnic group on the 2011 Census, but recent estimates put the Bristol Somali population at 10,000 [37, 38] and over 5% (1 in 20) of all school children in Bristol are now Somali [38].

### Research characteristics and reflexivity

The project team consisted of two primary researchers (a PhD student and an undergraduate Psychology student, CL and SY), two Somali community researchers (AM and AF), and three qualitative methodology experts from the University (EC, SR, LB).

The two Somali researchers are from community organisations, one from the Somali Resource Centre (https://www.somalicentre.co.uk/) and one from Golden Key Bristol (http://www.goldenkeybristol.org.uk/). Both Somali researchers were involved in all aspects of the research, including: design and planning, producing materials, recruitment, analysis and contributing to this journal article. There was a pre-existing relationship between the University researchers and the Somali Resource Centre, as the centre had been involved in other research in the Bristol Somali community. Dissemination events will be planned to bring the findings from the project to the community.

Neither university researcher carrying out the focus groups identified as being from the Somali community; CL identifies as White British and SY as Chinese. This could have influenced the willingness of participants to talk openly about sensitive topics with those viewed as being outside of their community [35, 39]. Conversely participants also spoke about high levels of stigma from within their community, so the researchers being from outside the Bristol Somali community could have been a strength to enable discussion; by partnering with community organisations, the research team were able to build trust in the community during the project. CL had previously worked with members of the Somali community over a period of 18 months, while SY had no previous experience. In addition, the primary researchers (CL and SY) are both female and the Somali researchers (AM and AF) are both male. On discussion with the Somali community researchers (AM and AF), it was viewed as appropriate for the female primary researchers to lead the discussions in both the female and male focus groups as confidentiality was ensured within the groups and the study had the ‘endorsement’ of the community researchers for the participants to speak openly.

### Recruitment

The whole study team aimed to bring together a diverse group to explore different perspectives within the focus group setting [36]. Participants were recruited through Bristol Somali Resource Centre by advertising and word of mouth. Information sheets in both Somali and English were advertised at the Somali Resource Centre and the Somali researchers spoke to individuals as they used the facilities. Potential participants were asked if they knew anyone else who would be willing to take part in an attempt to reach out beyond users of the centre to include a wider sample of participants, though most participants were regular users of the resource centre. A diverse sample was sought in terms of age, gender, time in Bristol, number of children, and English language ability.

### Participants

For confidentiality in this close community, exact ages of participants were not collected by the research team, but in informal discussion with the participants they stated that they ranged in ages from late 20s to 60s, with the majority of participants aged between 30 and 45. The participants had been living in Bristol between less than a year to over 20 years and had a wide range of verbal English language skills, from very basic to advanced/fluent. CL and SY, along with the Somali community partners, did not formally assess English language skills, but asked participant preference by offering the information sheet and consent form in English or Somali. The majority of participants had at least one child, but there was variation in the number and ages of participants’ children. This information was gained during informal discussion with the participants. Due to the close community, anonymous numerical codes were given to participants for presentation of illustrative quotes in the Results section.

### Ethical considerations

The study received ethical approval from the University of Bristol Faculty of Health Sciences Research Ethics Committee (FREC).

### Data collection

Focus groups were conducted at a community centre in Bristol as it was viewed as a neutral location that was convenient and socially acceptable to the study participants [35]. Participants were not given any contribution for taking part in the focus group, but refreshments were provided.

The whole study team, including the Somali community research partners (AM and AF) worked collaboratively to produce the study documents (participant information sheet, consent form and topic guides). The Somali community research partners (AM and AF) translated the English language participant information sheet and consent form into the Somali language, and took responsibility for focus group recruitment and providing translation services.

A topic guide covering mental health and access to healthcare was designed by the whole study team (Appendix) and consisted of broad questions serving as prompts to stimulate discussion. It was reviewed and revised as necessary after each focus group in relation to emerging data to ensure discussions covered all relevant topics.

The primary University of Bristol researchers (CL and SY) at the beginning of the focus group took informed consent from the participants. Participants were provided with the English and Somali versions of the information sheet and consent form and the community partners from the Bristol Somali Resource Centre and Golden Key Bristol checked understanding. Participants were informed that they could leave the group at any time, without having to give a reason.

On consultation with the Somali Resource Centre and Golden Key Bristol community partners, it was advised that it was culturally appropriate to hold separate focus groups for male and female participants to enable open discussion. Participants were thus invited to one of four focus groups with 5–6 people per group (2 female groups and 2 male groups). The focus groups lasted between 33 and 50 min. The focus groups were run by both primary researchers (CL and SY, with SY note taking [35]), with a Somali interpreter also present to provide continuous Somali to English translations throughout. Most participants spoke in Somali. Participants speaking English in the focus groups confirmed after translation that the translator had correctly relayed their own (or others) thoughts into English for the researchers. On a few occasions, study participants themselves translated for the researchers what others had said in Somali, so the researchers sought confirmation from the group translator that this was an accurate translation. This cross-checking between participants and the translator, of what was said in Somali, meant the researchers were confident in the English translations received during the focus groups [40]. The researchers ensured that all voices were heard during the focus group discussions by using strategies to engage those who seemed more hesitant, including direct questioning where necessary.

### Data analysis

The discussion was recorded on a voice recorder. CL and SY transcribed verbatim the English language translations provided by the interpreter in the focus groups. Names and personal information were removed to preserve anonymity.

Thematic analysis was used for data analysis [41, 42]. This involved initial coding, the forming and refining of categories, searching for negative evidence and constant comparison [43] across the data sets at each stage of the analysis. CL and SY used constant comparison to explore similarities and differences in the views of individuals emerging during focus groups [44, 45]. This enabled us to identify key themes and cases of divergence.

After the first two focus groups interviews, CL and SY each read the transcripts multiple times, independently, annotating the margin with general ideas, then draft ideas for ‘codes’ and then to group the ‘codes’ into broader ‘themes’. This was discussed and refined, and the codes and themes were agreed with the Somali community researchers. From this a thematic coding framework was drafted, which was then discussed with SR and the study team (AM, AF, EC, LB). NVivo software [46] was used for data management, and new codes from the final two focus groups were added to the coding framework. During this process, the coding framework was revised, merged and refined to develop a coherent thematic summary, which was discussed and agreed by the university researchers and the community partners. During coding, any links between codes was explored along with any negative evidence or contradictory aspects of the themes.

After the thematic summary was devised, the primary university researchers (CL and SY) and the Somali community researchers (AM and AF) met to discuss the findings and the recommendations for action from the results. These action items and ideas for future recommendations are presented in the discussion section.

## Results

### Participants

In total, 23 participants took part in the focus groups (12 male participants and 11 female). Four focus groups were held over 1 month in 2019. Focus groups 1 and 3 had female participants (six participants in group 1 and five participants in group 3) and focus groups 2 and 4 had male participants (six participants in each group).

No participants disclosed direct personal experiences with mental illness, but some participants made reference to personal experiences with family members, or others in the community.

Four key themes were identified during data analysis (Table 1): knowledge of mental illness; culture; ideas for improvements; and access to healthcare. These four themes will be discussed according to the male and female focus group perspectives and how this relates to young people in the Somali community in Bristol.

**Table 1:** Key themes from the focus groups

Theme	Sub Theme/s
1. Knowledge of mental illness	a. Lived Experiences b. Terminology c. Perceptions of the symptoms of mental illness
	d. Causes of mental illness
	e Treatment of mental illness
2. Culture	a. “Back in Somalia”
	b. Community Stigma
3.Improving mental health in the Bristol Somali community	
4. Access to healthcare	

#### Knowledge of mental illness

The first theme explores the participant’s knowledge of mental illness. Lived experiences and the terminology participants used to discuss mental ill health are illustrated, along with further subthemes of c) perceptions of the symptoms of mental illness, d) causes of mental illness and e) treatments of mental illness.

##### Lived experiences

In the female focus groups, none of the participants discussed a lived experience of mental illness, but participants did talk about others they have seen with mental illness.

“*I’ve seen people who are-the way that we know mental health is like someone who really really crazy*” (Female, FG3, P1)

“*but in here [Bristol] we haven’t seen to that stage that the person who is really crazy, running around the street…but they may have some kind of problem but not in an external that everybody can recognise that they have mental health*.” (Female, FG3, P1)

One male participant did talk openly in the group about negative personal experiences of a family member being sectioned with a mental illness in the UK and the perceptions of Somali individuals with mental illness by healthcare professionals:

“*the other thing is what people expect from the Somali individual in the ward, there, they, we have been referred as violent*” (Male, FG2, P4)

##### Terminology

Participants in the focus groups were asked about their knowledge of mental illness, including symptoms, causes and treatments. Although participants seemed to recognise (and most agreed) that there are different stages for the development of mental illness, participants described people in discrete categories, being either healthy or “*crazy*”/“mad” but accepted there may be hidden, non-visible, issues as well with individuals isolating themselves when experiencing symptoms.

“*So, there is mental healthy person, where the other person is mentally ill, is like someone who is totally crazy*” (Female, FG3, P6)

“*when you see him ‘oh he’s mad’ he can kill you and nobody can ask him about it.*” (Male, FG2, P3)

##### Perceptions of the symptoms of mental illness

Participants in the focus groups presented a spectrum of views on the symptoms a person might display when suffering from a mental illness. These ranged from the somatisation of symptoms and withdrawal from others to visible external displays of unusual behaviour.

Female focus group participants talked about and agreed that mental illness can start with physical symptoms

“*start with feeling headaches in generally”* (Female, FG1, P3)

Both male and female participants discussed that those with mental illness might isolate themselves, along with talking to themselves, not sleeping, or not dressing properly,

*“He is not probably dressing, he is probably a little bit violent.”* (Male, FG2, P2)

“*might isolate themselves. They might not trust anyone other than themselves”* (Female, FG3, P2)

Participants discussed how those with mental illness could display varied externalising behaviours from throwing things, to hurting others or even suicide:

*“They might reach a certain aim that they have to kill themselves or that they need hurt someone else”* (Female, FG3, P2)

*“Someone that hits the people. Someone who throws things and everything”* (Female, FG1, P2)

Females acknowledged that those experiencing mental illness might distance themselves from the rest of the community due to community reaction.

“*we always talk, the gossips*” (Female, FG3, P4)

But females recognised the negatives of isolation:

“*isolating the person always will kills them inside and then later the outside*.” (Female, FG3, P5)

However in one focus group, male participants discussed the distinction between those who display violent symptoms and those who do not.

“*mental health that cannot give you with problems in the community, it’s not violent, we know he’s got a mental health but it’s not going to give him any problems,*” (Male, FG2, P1)

When discussing mental health and young people, both female and male participants believed that young people would show the same symptoms as adults, since participants talked about mental illness as a universal issue.

*“The causes may be different from women to men, or from man to the young child, but he said [translating for other focus group participant] a problem is a problem in fact…”* (Male, FG4, P1)

*“Mainly mental health illnesses, diseases can affect at any stage and any age. So, it doesn’t have a specific age that the person might get…”* (Female, FG3, P1)

##### Causes of mental illness

Varied views as to the causes of mental illness were discussed during the focus groups with female and male participants offering different explanations.

Female participants talked about the main external cause of mental illness being family issues and stress, along with a lack of sleep and gender roles.

“*Maybe due to family issues, it starts with a little bit of stress*.” (Female, FG1, P2)

Family issues identified include children not coming home and a resulting lack of sleep.

“*lack of sleep is the main thing that causes*” (Female, FG1, P4 – with agreement from P’s 1 & 5)

“*mothers are the big issue, maybe fathers are always outside having kind of fun thing or at least chatting to another person, so that is why it is not that many big issues, but when it comes to the mothers the problems are bigger larger scale*” (Female, FG1, P1)

In contrast to the primary female view of mental illness being caused by family issues, male participants attributed mental ill health to economic reasons.

“*The triggers of that are something to do with the economic collapse*” (Male, FG2, P3)

“*you want to successful and you try successful and you losing all the times”* (Male, FG2, P5)

Male participants gave examples of potential economic issues that may cause mental issues, such as

- poverty: “*poverty is another thing*” (Male, FG2, P4),
- unemployment: “*when you have no jobs that’s one of the worst*” (Male, FG2, P5),
- and homelessness: “*if you are unemployed um homeless that’s it*” (Male, FG4, P4).

Trauma was also recognised as a potential cause by male participants.

“*back home when there was civil war, because of that environment they were in they could get some form of mental health symptoms*” (Male, FG2, P1)

A few male participants also mentioned family separation and family conflict, including mothers being unable to communicate with children. Here views revealed some similarities with those held by the female participants.

“*There is a family breakdown and a language gap*” (Male, FG2, P3)

“*Mothers having problem and mothers having problem with the father because father he can go out a little bit and relax go to the cafeteria and can talk to other people, or the Mosque, but mother she is at home*” (Male, FG4, P6)

“*So mental problems starting from the house*” (Male, FG4, P6)

In terms of common mental illnesses, anxiety and depression were viewed as “*a part of life*,” (Male, FG4, P5)

“*One week you are-got anxiety depression. One week you are very happy, the other week…It’s a part of the life*” (Male, FG4, P4)

“*we don’t think that’s a mental health*” (Male, FG2, P3)

“*we view mental health as someone who just throws away his clothes, or someone killing people, something like that*” (Male, FG2, P3)

One participant disagreed and suggested that “*before you get a serious mental health, it starts from depression and anxiety*” (Male, FG4, P1), illustrating a potential escalation perception of mental illness, starting with the hidden and then leading to more visible, ‘crazy’ external behaviours.

Other participants referred to the ‘crazy’ category when talking about anxiety and depression.

“*it might be someone who just a little bit depressed or a little bit anxiety. In Somali yeah, people they stressed, talked and asking who is sick, she is crazy, she is crazy*” (Female, FG1, P2)

In terms of young people, female participants talked about how mental health issues might be heritable in children as there is a belief that if the oldest child suffers mental ill health, the other children might follow. But some female participants also stated that this might be due to children imitating other children’s abnormal behaviours.

“*I don’t know if something to do with being heritability unstable for all of them, or they are just trying to copy that-the eldest person is sick and the way he or she behave*.” (Female, FG3, P6)

Participants also talked about how young people might not recognise mental illness in themselves until they grow older.

“*Early years the teenagers or the kids they might not understand what they are feeling. But as you get older, you may know you have something, some problem with you*” (Female, FG3, P2)

Also, socialising and feelings of belonging, were discussed by male participants in relation to young people:

“*they don’t have the communities, they don’t have anywhere to socialise, so that is for them is a mental health trigger*,” (Male, FG4, P3)

##### Treatments of mental illness

Participants discussed how individuals displaying symptoms of mental illness could be treated within their community.

Female participants stated that they would look to religious healing for the treatment of mental illness, such as reading the Qur’an. Female participants were also aware of medication and that talking to others might help improve mental illness.

Men also stated religious healing would be used, but also medications, institutions and taking the person out of the country could be used as potential treatments for mental illness.

“*we go to a Mosque and read the Qur’an*.” (Male, FG4, P3)

“*we felt like NHS do not understand what we want, we sent my [family member] back home and he’s ok, thanks God*” (Male, FG2, P4)

#### Culture

Participants in the focus groups discussed the unique cultural aspects of mental illness and mental health in the Bristol Somali community.

##### “Back in Somalia”

Both male and female participants talked about the trauma experienced from the Civil War in Somalia and how this may impact UK Somali communities, along with cultural disconnect.

“*our body is here but our brain and thoughts and thinking are in Somalia*” (Male, FG4, P3)

Male participants talked about how those with mental illness would have been treated in Somalia and a lack of awareness.

“*we are not aware about mental health when we were back home*.” (Male, FG2, P3).

##### Community stigma

In the Bristol Somali community, participants talked about how those with mental illness are viewed as “*crazy*” and how mental illness is a taboo subject in the community.

“*it’s embarrassing for the families to admit we need help*” (Male, FG2, P4)

“*there are so many people amongst Somali community, it’s a really big taboo subject*.” (Female, FG1, P3)

Female participants said that they were “*afraid*” (Female, FG3, P1 – with agreement from Pt’s, 2 & 3) of talking to those with mental health issues and tried to avoid them. In line with views and symptoms of mental illness discussed previously, participants also gave accounts of how ‘community gossip’ led to those with mental illness to hide away from public gaze.

Female participants went on to discuss how they would also be afraid of young people with mental illness, as they believed that age was not a relevant factor and that mental illness in young people is viewed with the same stigma as adults.

“*Crazy person is crazy person. It doesn’t differentiate. It’s always the same if it’s a child, and or small kid whatever they are. They just, for us it’s always the crazy person, the crazy person, that’s it*.” (Female, FG3, P6)

#### Improving mental health in the Bristol Somali community

Both male and female participants identified that increased community support and services would be helpful for those experiencing mental illness. Most male and female participants recognised there has been an increase in places to get support.

“*Nowadays there are so many charities that are helping*” (Female, FG3, P3)

Female participants stressed the importance of education, training and raising awareness in how to help those with mental ill health.

“*what we need to understand is like, mental health [problems] is not just about craziness*” (Female, FG1, P2)

“*it would have been so good if they have those advocates, those trainings, that they can really give the woman back to the community*.” (Female, FG1, P4)

Other female participants echoed this view:

“*Awareness is the first and the most, educating the community, understand that this is an illness. It might-it doesn’t have-it doesn’t discriminate which age or which group of sex you are on*.” (Female, FG3, P4)

Men identified that increased services and having elder Somali staff to talk to would be useful if a person did not want to talk to their GP, along with a triage system and care in the community.

“*it would make sense if you could talk to some Somali person who could understand you rather than going to your GP*” (Male, FG2, P4)

“*Mental health team to recruit an elderly, an elderly person who understands the culture of the community*.” (Male, FG2, P3)

“*we need a system who can easily find out people who are experiencing mental health [problems], who have seriously got the mental health [problems] so that can be treated very quickly”* (Male, FG2, P1)

Some male participants also identified English language education for women, in order to communicate better with their children, in relation to perceived causes of mental illness, rather than as an idea to improve service uptake. In terms of young people, increased facilities and community groups were identified as helpful.

“*who are round the city and just standing doing nothing…even those kids have some kind of mental health problem*” (Female, FG1, P2)

#### Access to healthcare

The fourth theme evident from the focus groups was discussions on accessing healthcare.

Women were generally happy with the services they received from medical professions and viewed GPs as “*the backbone of everything*” (Female, FG1, P6).

“*we don’t have, not that many barriers affecting with the health services. Whatever problem we have in mind, the first person to contact will always be the GP*.” (Female, FG3, P2)

But some female participants did voice problems with accessing healthcare, including: being unsure where to access services, language barriers, long waiting times and a lack of continuity with seeing different medical professionals each time they visited.

*“You can’t seek help outside and you don’t know how to approach that person who is not in your language speaking”* (Female, FG1, P1)

*“it’s always good to seek help but we don’t know how to and that’s the main thing”* (Female FG1, P2)

On making a GP appointment: *“You have to wait 2 weeks, 3 weeks, sometimes 4 weeks…”* (Female, FG1, P4)

*“Every time you make an appointment you see a different GP which is a big problem.”* (Female, FG3, P4)

Female participants talked about the interpreter available in their local GP service “*having an interpreter stand by is always good…where you have access someone here being from 9 to 5 is always a big point. Yeah well done to the* [GP surgery name]” (Female, FG1, P4).

Most male participants echoed the views of females, but one male participant voiced his mistrust of perceived authority figures and fear of going to the doctor or getting treated due to potential economic repercussions and past experiences of a family member in the community.

*“There are not many, there’s nowhere to turn other than locally, apart from going to your doctors which is a part of what we are so afraid of.”* (Male, FG2, P4)

*“I won’t tell you my problem, simple, that’s the problem*” (Male, FG2, P4)

*“we don’t wanna go into those institutions*” (Male, FG2, P4)

“*we don’t like the police because they intimidate you, they put you down for no reason and health services same as that*” (Male, FG2, P4)

“*your GP who will later on section your driving licence for God’s sake if you tell them you haven’t slept the last few days*” (Male, FG2, P4)

“*rather than the doctor penalising you*” (Male, FG2, P4).

This view was not shared by all the focus group, although there was some agreement.

Other male participants talked about practical issues accessing primary healthcare, but recognised this was a UK wide issue, not specific to Somali communities.

“*in the UK they have problem with GP, we are part of that. So, in GP for the appointment to see the doctor, it takes long time. So that is not for us only, for the UK problem ok*” (Male, FG4, P6).

But secondary healthcare was viewed more positively: “*When you are in the primary care setting, it will be difficult to access a proper treatment. But when you go to the secondary care, it’s very easy*” (Male, FG4, P5)

Both females and males stated that they would always go to the doctor for their child, although there is potentially some male distrust of the GP.

*“I will take the young person to the GP and discuss, the adult person never go…”* (Male, FG2, P1)

Male participants also talked about different types of young people – those who were born in the UK and those who moved to the UK. Fluency in English and poor Somali language skills however could also have the potential of causing higher levels of mental illness due to lack of communication with the family as previously discussed.

“*there are different types of young people, the young generation those who born here they can manage themselves because they are part of the community, but those who came here as an adult then they have some problems because to integrate other people or to stay with their community…*” (Male, FG4, P6)

## Discussion

In this study we explored Somali views on mental illness and accessing health care services to identify the perspectives of mental illness amongst a UK Somali community with an aim to improve mental health services. The most important community views about the barriers to accessing mental health care for men, women and children were: knowledge of mental illness; culture; and access to health care in general. In addition, the community provided ideas for improvements in mental illness recognition and accessing culturally safe support services for the British Somali community. Culture was explored in depth in the focus groups and was a key theme that could shape the illness beliefs in this community.

In our study, Somali women explained that mental illness and feelings of distress start with, and would be described to a GP as a “headache”. This illustrates the conceptualisation of mental illness symptoms in the Somali community, which may differ from mainstream illness beliefs. Headaches are a physical symptom that has been associated with prolonged psychological distress in Somali adults in the USA [47] and physical symptoms of mental illness have been described in Somali adults, such as dizziness, poor vision and heat coming out of the head [10]. In this study the fact that some Somali females explained that mental illness starts with the physical symptom of headaches is a new finding and to our knowledge has not been described. This is important clinically, as the presentation of headaches in females could be a descriptor for stress or mental health issues. In this context, the somatisation of mental illness symptoms could be a protective mechanism as somatisation is common amongst migrants, and higher rates of somatisation have been linked to higher rates of mental distress [48]. Somatisation provides challenges for healthcare professionals in terms of social, linguistic and cultural differences [48]. GPs and primary care providers may need additional awareness training on how mental illness and symptoms of distress manifest in different communities.

Further findings in the present study are different accounts of how mental illness may be caused. Female participants attributed mental illness mainly to family issues while males mainly referred to economic reasons (e.g. employment, homelessness) as a cause of mental ill health. Previous research has found high levels of unemployment in the Somali communities in the UK [49–52] consistent with the findings from this study and the different attributions for the causes of mental illness could be due to traditional Somali gender roles [49]. Participants also discussed previous trauma from the civil war as a potential cause of mental ill health due to PTSD symptoms and migration experiences [5, 6].

The causes of mental illness in children were discussed as being heritable or imitating the behaviour of another child with a mental illness. This was explained by a belief that if the first child has mental health issues, the other children might also experience symptoms due to heritability or imitation. This is an interesting finding, which to our knowledge has not been described before. In the Bristol Somali community, there have been campaigns to improve awareness of autism in children [29, 34, 53]. Due to the heritable nature of autism [54], and the impact on siblings of having a sibling with a developmental disorder [55, 56], this could provide an explanation for the belief found in this study that mental illness is heritable or imitation. Healthcare professionals need to be aware of the belief that if one child in the family is diagnosed with a developmental disorder or a mental illness, other children may also be susceptible.

In the focus groups, participants discussed that mental illness could be exacerbated by a cultural gap between parents and children, based on life experiences. This cultural gap has been found in literature looking at Somali adolescents and mental health previously [12]. Participants in this project identified a further distinction between children born in the UK and those who moved to the UK. Participants described fewer barriers in the former group (those born in the UK) in terms accessing healthcare, due to English language ability and understanding the UK healthcare system. Female participants perceived a person to be either healthy or “crazy” and stated that this distinction is also the case in children who may be experiencing distress. They would avoid an individual of any age that displayed mental illness due to the stigma and an expectation of violent behaviour.

In terms of treatment for mental illness, males suggested that removing a person suffering from mental ill health from the UK and returning to Somalia could be viewed as a treatment option to receive culturally appropriate care. Or alternatively, returning to Somalia could be due to a distrust of Western medical institutions and the use of psychiatric medication [16], along with negative views in the community about inpatient mental health care in ‘institutions’. Since 2003, evidence has shown that Black British males in the UK are more likely to detained under the Mental Health Act [19], and experience higher rates of compulsory in-patient treatment for mental illness compared to White British males [57]. These higher rates of detention and inpatient treatment could potentially be a reason for the mistrust. Male participants also discussed GPs as authority figures and expressed a worry of repercussions from accessing treatment for mental health along with how mental health problems could add to economic hardship. Potential solutions currently being piloted in London to improve trust and access to mental health services for ethnic minority individuals include teaching modules, Mental Health Champions in the community and the inclusion of Faith Consultants [58]. In Bristol, numerous charitable organisations are providing support and engaging individuals suffering from mental illness by providing individuals with a choice over how they engage with tailored support. GPs and healthcare professionals should be aware of this continued, mistrust, especially among males, and the categorisation and stigma of mental illness in the Somali community along with concerns about repercussions for accessing treatment.

In addition to the novel findings in our study, other results are consistent with previous research findings in Somali communities on mental health and illness and strengthen the evidence base. Stigma can play a role in any individual accessing mental health services [59], but cultural stigma in close BAME communities, such as the Somali community in Bristol, has been described previously as a key barrier in accessing mental health services [7, 8, 12, 16]. Participants in the current study also discussed the importance of religion and for religious principles and practices to be incorporated into treatment plans [7, 12, 16]. Furthermore, participants in the focus groups supported previous findings that mental illness is only identified in scenarios when individuals display noticeable behaviours which are categorised as “crazy”, such as removing clothing [17, 60], although some participants in the current study talked about a ‘sliding scale’ starting with problems like depression and anxiety which escalates to observable aberrant behaviour. Other participants viewed depression and anxiety as a part of life [60]. Different words can be used to describe Somali expressions of mental distress; one can be translated into English and defined as ‘sadness’ which is on a sliding scale from everyday stress to more serious depression that could cause ‘craziness’, and one which is more stigmatised and defined as ‘crazy’ [47]. Therefore different terminology can be used to describe distress ranging from everyday stress or sadness, to visible symptoms defined as ‘crazy’ [47].

The present study investigated access to medical care in this community with the majority of participants expressing satisfaction with accessing medical care and the medical care that they received. Women talked about an interpreter being available for appointments but mentioned barriers in terms of long waiting times and seeing different medical professionals on each visit, issues which are UK wide and not specific to BAME individuals. Men echoed this viewpoint, but some voiced a reluctance of going to the doctor due to the perception of the GP as an authority figure and a distrust of authority, which may be related to previous adverse experiences with people in positions of power. The NHS has identified that adverse experiences of hospital mental health services is a cause for concern among BAME groups which can lead to mistrust of services [20]. In our study, participants were clear that they would always take a child to the GP, despite any mistrust towards the doctors, as children born in the UK may have more understanding of the health care system and different English language abilities to their parents. This finding is similar to previous work in a BAME population, which found that parents had more positive attitudes towards seeking healthcare for their children than they would for themselves and perceived fewer and different barriers and more positive attitudes for their children receiving care than themselves [61].

Based on their experience, our community partners expressed surprise that participants did not report more access to healthcare barriers. There are several possible explanations for this: only those who were generally happy with the healthcare they had received agreed to take part in our focus groups, or the participants might have some issues with healthcare but did not feel it was appropriate to either disagree with other participants who viewed healthcare positively [35, 62]. Alternatively, the participants may not have wanted to discuss with the University researchers any issues they might have faced when accessing healthcare due to cultural mistrust [39]. It should be noted that the local GP surgery in the area where the participants were recruited from has provided considerable resources to those who need them, including having interpreters on standby and providing community outreach services. This can be considered relatively unique to this GP practice and could have influenced the participants’ positive responses.

### Recommendations from the study participants and Somali researchers

Participants in the study had varied ideas on how to improve knowledge and understanding of mental illness and how to improve access to services. Males thought having Somali staff to talk to would be useful if an individual did not want to talk to a GP. This supports previous literature which found that Somalis would trust a Somali staff member more than a non-Somali [7]. Increased facilities and community social groups in the local area for Somali families and young people, with a focus on social activity and to give young people and teenagers a place to meet and socialise, were also suggested as helpful. Participants in the focus groups noted that young people and teenagers have limited options for places to go for social activities and this was viewed as a risk factor for mental health. This supports previous studies investigating mental health in BAME young people in which more social activity for young people was suggested, e.g. youth clubs [63].

Increased community support and community services were seen as valuable by the study participants, along with education for training and awareness to help those with mental ill health and to reduce stigma. In particular, participants thought training should include education to reduce the stigma of ‘craziness’ associated with mental illness, increase understanding of mental health symptoms and the ways in which mental illness presents, along with places to access help. The community partners also endorsed the ideas from the participants in the focus groups of workshops and knowledge building events to provide education on mental illness and to challenge and reduce the stigma of mental illness within the Somali community in Bristol.

The community partners also suggested that mental health services could increase community based services through the utilisation of social prescribing [64] or Health Link workers to develop trust and relationships between community members and mental health services. Social prescribers can be referred to as Link Workers [65] or link workers can be qualified CBT therapists or wellbeing advisors [66]. Social prescribing is an intervention based in the community that links primary care patients with community support, including cultural, social and leisure activities [67, 68]. Social prescribing has proven mental health benefits to service users [67] and can link patients with health resources and social support independent of the NHS [68].

GPs and the NHS are increasingly recognising that social prescribing is valuable, and community resources are important in supporting patients’ health with the NHS employing social prescribers since 2019 [65, 69]. Utilising social prescribing and health link workers would take a holistic approach to people’s wellbeing and lead to a deeper understanding of social factors that exacerbate mental illness, and cultural treatment preferences that consider for example the role of religion. As health link workers can be employed to raise awareness of common mental disorders in BAME groups, and how mental health services can improve symptoms [66], the use of health link workers could provide knowledge building resources to the Bristol Somali community.

### Strengths and limitations

Due to the community partnership, the researchers were able to hold focus groups with hard to reach participants; those who cannot speak English fluently or would prefer to communicate in Somali, and those who would be hesitant to participate in the research without the community partner’s involvement. In addition, though the use of CBPR methodology, the Somali researchers were involved in the analysis of the data through discussing and agreeing the ‘codes’ and ‘themes’, along with consensus on the coding framework, and providing input into how to interpret and report the results of the study. This ensures the results and recommendations from the study are culturally acceptable and relevant due to the unique insight into the study findings that the Somali community partners could provide due to their knowledge of the Bristol Somali community.

Focus groups were chosen to enable participants to discuss their views within a group setting [36]. At the beginning of the focus groups, the researchers made it clear that the discussion was confidential and should stay in the room. The researchers did everything possible to try and enable all participants to voice their views, but in one focus group, a male identified as a “leader” in Somalia, which may have changed the dynamics of the group, since other participants may have been reluctant to disagree with the views of a leader [35, 36] or to express an alternative view [62]. Future studies could follow up with one-on-one interviews to further strengthen the evidence base by including personal narratives of mental illness and accessing help within this group.

The participants recruited for the focus groups depended on those willing and able to attend the focus groups. The researchers aimed for this project to develop local solutions to a problem identified by the community, but are aware that not all views could be included in the project. The researchers attempted to mitigate this by ensuring a good representation from the community, recruiting participants with a wide range of ages, number of children, time spent living in the UK and English language ability [39] to develop rigorous solutions to the problem identified.

The researchers CL and SY relied on a running translation in the focus groups from Somali to English. Nearly all participants chose to speak Somali which could have affected the ability of the researchers to guide the discussion in response to participant comments [70]. The researchers used the same male translator for both male groups and the same female translators for both female groups to aim to maximise the reliability of the study data [71].

The current study was carried out in Bristol so this data represents the views of participants in one geographic area, as most participants lived in one neighbourhood. Also, this research was carried out in a country where healthcare is free to all citizens/residents at the point of access. Different barriers to accessing healthcare may be found in a country without universal health coverage, free at the point of access.

## Conclusions

This study makes an important contribution to the literature and has highlighted the conceptualisation of mental health and mental illness in the Somali community in Bristol, which may differ from mainstream illness beliefs. The study results give rise to ideas for practice that may be relevant to GPs, health professionals, charities and those working with the UK Somali community. There may be different conceptualisations of symptoms in the Somali community with somatisation of psychological problems, for example females may refer to headaches and stress. There are also varied reasons attributed to the causes of mental illness, which professionals need to be aware of along with the stigma of mental illness in this community. The utilisation of social prescribing or health link workers could be implemented to develop relationships and trust between the Somali community and health care providers, along with community knowledge building and awareness events.

## Data Availability

Not applicable.

## Appendix

### Topic guide

This topic guide details focus group topics to be covered.

The researchers might also ask additional questions to clarify information.

#### Prior to the start of the focus group

The aim of this study is to understand your views on mental illness and mental ill health within the Somali community in Bristol.

This focus group will be recorded and during transcription any details, which could identify yourself or others, will be made confidential. You can ask to stop or you can leave this focus group at any time without having to give a reason.

#### Part A: knowledge of mental health

- Tell me what you think about mental health
- Prompts:
  - Beliefs about seeking mental health care
  - Describing mental health
  - Language – do you know what depression/anxiety is?
  - Views on hidden healthcare conditions
  - Views on a person who doesn’t get out of bed/talks about fatigue/tiredness

#### Part B: accessing health care services

- Tell me about accessing healthcare
- Prompts:
  - Factors influencing healthcare seeking
  - Relationship with GP/medical services
  - Seeking help from places other than GP

#### Part D: information about the community

- Tell me about the community’s views on mental health
- Prompts:
  - Community barriers / influences on accessing healthcare services

#### Part E: close

- Are there any issues you would like to raise that we have not talked about?
- Thank you for your time and contribution to this study

#### Prompts: the focus group facilitators (to explore certain answers in more detail) may also use the following prompts

- Can you describe that?
- Could you give me an example?
- Could you tell me more about that?

## References

[1] McManus S, Bebbington P, Jenkins R, Brugha T (2016). Mental health and wellbeing in England: adult psychiatric morbidity survey 2014.

[2] NHS Digital. Mental health of children and young people in England: NHS Digital; 2017. Available from: https://digital.nhs.uk/data-and-information/publications/statistical/mental-health-of-children-and-young-people-in-england/2017/2017. Cited 2019 Oct 7.

[3] McGorry PD, Mei C (2018). Early intervention in youth mental health: progress and future directions. Evid Based Ment Health.

[4] Edwards J, Anderson K, Stranges S (2019). Migrant mental health, Hickam’s dictum, and the dangers of oversimplification. Int J Public Health.

[5] Onyut LP, Neuner F, Ertl V, Schauer E, Odenwald M, Elbert T (2009). Trauma, poverty and mental health among Somali and Rwandese refugees living in an African refugee settlement – an epidemiological study. Confl Heal.

[6] Palmer D, Ward K (2007). ‘Lost’: listening to the voices and mental health needs of forced migrants in London. Med Confl Surviv.

[7] Wolf KM, Zoucha R, McFarland M, Salman K, Dagne A, Hashi N (2016). Somali immigrant perceptions of mental health and illness: an ethnonursing study. J Transcult Nurs.

[8] Memon A, Taylor K, Mohebati LM, Sundin J, Cooper M, Scanlon T (2016). Perceived barriers to accessing mental health services among black and minority ethnic (BME) communities: a qualitative study in Southeast England. BMJ Open.

[9] Hammond L (2013). Somali transnational activism and integration in the UK: mutually supporting strategies. J Ethn Migr Stud.

[10] Bettmann JE, Penney D, Freeman PC, Lecy N (2015). Somali refugees’ perceptions of mental illness. Soc Work Health Care.

[11] McCrone P, Bhui K, Craig T, Mohamud S, Warfa N, Stansfeld SA (2005). Mental health needs, service use and costs among Somali refugees in the UK. Acta Psychiatr Scand.

[12] Ellis BH, Lincoln AK, Charney ME, Ford-Paz R, Benson M, Strunin L (2010). Mental health service utilization of Somali adolescents: religion, community, and school as gateways to healing. Transcult Psychiatry.

[13] Palmer D (2006). Imperfect prescription: mental health perceptions, experiences and challenges faced by the Somali community in the London Borough of Camden and service responses to them. Prim Care Ment Health.

[14] Loewenthal D, Mohamed A, Mukhopadhyay S, Ganesh K, Thomas R (2012). Reducing the barriers to accessing psychological therapies for Bengali, Urdu, Tamil and Somali communities in the UK: some implications for training, policy and practice. Br J Guid Couns.

[15] Markova V, Sandal GM (2016). Lay explanatory models of depression and preferred coping strategies among Somali refugees in Norway. A mixed-method study. Front Psychol.

[16] Whittaker S, Hardy G, Lewis K, Buchan L (2005). An exploration of psychological well-being with young Somali refugee and asylum-seeker women. Clin Child Psychol Psychiatry.

[17] Guerin B, Guerin PB, Diiriye RO, Yates S (2004). Somali conceptions and expectations concerning mental health: some guidelines for mental health professionals. N Z J Psychol.

[18] Boynton L, Bentley J, Jackson JC, Gibbs TA (2010). The role of stigma and state in the mental health of Somalis. J Psychiatr Pract.

[19] Gajwani R, Parsons H, Birchwood M, Singh SP (2016). Ethnicity and detention: are Black and minority ethnic (BME) groups disproportionately detained under the Mental Health Act 2007?. Soc Psychiatry Psychiatr Epidemiol.

[20] Public Health England (2018). Health matters: reducing health inequalities in mental illness - GOV.UK.

[21] Halvorsrud K, Nazroo J, Otis M, Brown Hajdukova E, Bhui K (2018). Ethnic inequalities and pathways to care in psychosis in England: a systematic review and meta-analysis. BMC Med.

[22] Kirkbride JB (2018). Addressing ethnic inequalities in the pathways to care for psychosis. BMC Med.

[23] Wessley S (2018). Modernising the mental health act – final report from the independent review.

[24] National Institute for Mental Health in England. Inside outside: improving mental health services for black and minority ethnic communities in England. Leeds: Department of Health; 2003.

[25] Bristow K, Edwards S, Funnel E, Fisher L, Gask L, Dowrick C (2011). Help seeking and access to primary care for people from “hard-to-reach” groups with common mental health problems. Int J Fam Med.

[26] Department of Health, UK (2014). Achieving better access to mental health services by 2020.

[27] Mclean C, Campbell C, Cornish F (2003). African-Caribbean interactions with mental health services in the UK: experiences and expectations of exclusion as (re) productive of health inequalities. Soc Sci Med.

[28] Warfa N, Bhui K, Craig T, Curtis S, Mohamud S, Stansfeld S (2006). Post-migration geographical mobility, mental health and health service utilisation among Somali refugees in the UK: a qualitative study. Health Place.

[29] Fox F, Aabe N, Turner K, Redwood S, Rai D (2017). “It was like walking without knowing where I was going”: a qualitative study of autism in a UK Somali migrant community. J Autism Dev Disord.

[30] Minkler M, Wallerstein N, editors. Community-based participatory research for health: from process to outcomes. New York: Wiley; 2011.

[31] Johnson CE, Ali SA, Shipp MP-L (2009). Building community-based participatory research partnerships with a Somali refugee community. Am J Prev Med.

[32] Wright C, Wright S, Diener M, Eaton J (2014). Autism spectrum disorder and the applied collaborative approach: a review of community based participatory research and participatory action research. J Autism.

[33] Cargo M, Mercer S (2008). The value and challenges of participatory research: strengthening its practic. Annu Rev Public Health.

[34] Selman LE, Fox F, Aabe N, Turner K, Rai D, Redwood S (2018). ‘You are labelled by your children’s disability’ – a community-based, participatory study of stigma among Somali parents of children with autism living in the United Kingdom. Ethn Health.

[35] Halcomb EJ, Gholizadeh L, DiGiacomo M, Phillips J, Davidson PM (2007). Literature review: considerations in undertaking focus group research with culturally and linguistically diverse groups. J Clin Nurs.

[36] Kitzinger J (1995). Qualitative research: introducing focus groups. BMJ.

[37] Bristol City Council (2019). The population of Bristol.

[38] Mills J. Bristol City Council: community profile Somalis living in Bristol: Bristol City Council; 2014. Available from: https://www.bristol.gov.uk/documents/20182/33107/Equality+Profile+Somalis+2014.pdf/99f13ec0-da03-4928-9971-77246a812b17.

[39] Blake HM, Irvine ST (2003). Challenges and strategies for conducting survey and focus group research with culturally diverse groups. Am J Speech Lang Pathol.

[40] Plumridge G, Redwood S, Greenfield S, Akhter N, Chowdhury R, Gill P (2012). Involving interpreters in research studies. J Health Serv Res Policy.

[41] Braun V, Clarke V (2006). Using thematic analysis in psychology. Qual Res Psychol.

[42] Braun V, Clarke V. What can “thematic analysis” offer health and wellbeing researchers? Int J Qual Stud Health Well-being. 2014;9(1). 10.3402/qhw.v9.26152. 10.3402/qhw.v9.26152 PMC4201665 25326092

[43] Glaser BG (1965). The constant comparative method of qualitative analysis. Soc Probl.

[44] Onwuegbuzie AJ, Dickinson WB, Leech NL, Zoran AG (2009). A qualitative framework for collecting and analyzing data in focus group research. Int J Qual Methods.

[45] Leech NL, Onwuegbuzie AJ (2011). Beyond constant comparison qualitative data analysis: using NVivo. Sch Psychol Q.

[46] Richards L (2016). NVivo qualitative data analysis software version 11.0. Released.

[47] Carroll JK (2004). Murug, waali, and gini: expressions of distress in refugees from Somalia. Prim Care Companion J Clin Psychiatry.

[48] Lanzara R, Scipioni M, Conti C (2019). A clinical-psychological perspective on somatization among immigrants: a systematic review. Front Psychol.

[49] Warfa N, Curtis S, Watters C, Carswell K, Ingleby D, Bhui K (2012). Migration experiences, employment status and psychological distress among Somali immigrants: a mixed-method international study. BMC Public Health.

[50] Evans D, Page D (2012). ‘Somali’ refugees, mental health and employability in the Southwest: exploring the barriers to inclusion.

[51] Khan K (2008). Employment of foreign workers: male and female labour market.

[52] Jabłonowski J, Mohamed A, Sugulle A. Work and health of Somali migrants in Bristol. Bristol: Wellspring Health Living Centre and Bristol City Council; 2013.

[53] NIHR ARC West. Overcoming barriers: a short film to promote culturally sensitive services to support Somali families affected by autism. Available from: https://arc-w.nihr.ac.uk/research/projects/a-short-film-to-promote-culturally-sensitive-services-to-support-somali-families-affected-by-autism/. Cited 2020 Jul 9.

[54] Sandin S, Lichtenstein P, Kuja-Halkola R, Larsson H, Hultman CM, Reichenberg A (2014). The familial risk of autism. JAMA.

[55] Petalas MA, Hastings RP, Nash S, Lloyd T, Dowey A (2009). Emotional and behavioural adjustment in siblings of children with intellectual disability with and without autism. Autism.

[56] Griffith GM, Hastings RP, Petalas MA (2014). Brief report: fathers’ and mothers’ ratings of behavioral and emotional problems in siblings of children with autism spectrum disorder. J Autism Dev Disord.

[57] Bhui K, Stansfeld S, Hull S, Priebe S, Mole F, Feder G (2003). Ethnic variations in pathways to and use of specialist mental health services in the UK: systematic review. Br J Psychiatry.

[58] Codjoe L, Barber S, Thornicroft G (2019). Tackling inequalities: a partnership between mental health services and black faith communities. J Ment Health.

[59] Rüsch N, Angermeyer MC, Corrigan PW (2005). Mental illness stigma: concepts, consequences, and initiatives to reduce stigma. Eur Psychiatry.

[60] Piwowarczyk L, Bishop H, Yusuf A, Mudymba F, Raj A (2014). Congolese and Somali beliefs about mental health services. J Nerv Ment Dis.

[61] Thurston IB, Phares V (2008). Mental health service utilization among African American and Caucasian mothers and fathers. J Consult Clin Psychol.

[62] Clark MJ, Cary S, Diemert G, Ceballos R, Sifuentes M, Atteberry I (2003). Involving communities in community assessment. Public Health Nurs.

[63] Ali N, McLachlan N, Kanwar S, Randhawa G (2017). Pakistani young people’s views on barriers to accessing mental health services. Int J Cult Ment Health.

[64] South J, Higgins T, Woodall J, White S (2008). Can social prescribing provide the missing link?. Prim Health Care Res Dev.

[65] Drinkwater C, Wildman J, Moffatt S (2019). Social prescribing. BMJ.

[66] Evans L, Green S, Sharma K, Marinho F, Thomas P (2014). Improving access to primary mental health services: are link workers the answer?. London J Prim Care.

[67] Wahlbeck K, Cresswell-Smith J, Haaramo P, Parkkonen J (2017). Interventions to mitigate the effects of poverty and inequality on mental health. Soc Psychiatry Psychiatr Epidemiol.

[68] Kimberlee R. What is social prescribing? Adv Soc Sci Res J. 2015;2(1):102–10.

[69] Cawston P (2011). Social prescribing in very deprived areas. Br J Gen Pract.

[70] Esposito N (2001). From meaning to meaning: the influence of translation techniques on non-English focus group research. Qual Health Res.

[71] Twinn S (1997). An exploratory study examining the influence of translation on the validity and reliability of qualitative data in nursing research. J Adv Nurs.

